# FUNDC1: A Promising Mitophagy Regulator at the Mitochondria-Associated Membrane for Cardiovascular Diseases

**DOI:** 10.3389/fcell.2021.788634

**Published:** 2021-12-16

**Authors:** Guoyong Li, Junli Li, Ruochen Shao, Jiahao Zhao, Mao Chen

**Affiliations:** ^1^ Laboratory of Heart Valve Disease, West China Hospital, Sichuan University, Chengdu, China; ^2^ Department of Cardiology, West China Hospital, Sichuan University, Chengdu, China; ^3^ West China School of Medicine, Sichuan University, Chengdu, China

**Keywords:** FUNDC1, mitophagy, cardiovascular diseases, LC3, MAM

## Abstract

Mitochondrial autophagy (or mitophagy) regulates the mitochondrial network and function to contribute to multiple cellular processes. The protective effect of homeostatic mitophagy in cardiovascular diseases (CVDs) has attracted increasing attention. FUN14 domain containing 1 (FUNDC1), an identified mitophagy receptor, plays an essential role in CVDs. Different expression levels of FUNDC1 and its phosphorylated state at different sites alleviate or exacerbate hypoxia and ischemia/reperfusion injury, cardiac hypertrophy, or metabolic damage through promotion or inhibition of mitophagy. In addition, FUNDC1 can be enriched at contact sites between mitochondria and the endoplasmic reticulum (ER), determining the formation of mitochondria-associated membranes (MAMs) that regulate cellular calcium (Ca^2+^) homeostasis and mitochondrial dynamics to prevent heart dysfunction. Moreover, FUNDC1 has also been involved in inflammatory cardiac diseases such as septic cardiomyopathy. In this review, we collect and summarize the evidence on the roles of FUNDC1 exclusively in various CVDs, describing its interactions with different cellular organelles, its involvement in multiple cellular processes, and its associated signaling pathways. FUNDC1 may become a promising therapeutic target for the prevention and management of various CVDs.

## 1 Introduction

Cardiovascular diseases (CVDs), as a constant public health burden, are the leading cause of morbidity and mortality worldwide. CVD-related mortality has been reduced due to initiative prevention and pharmaceutical and technological improvements. However, the CVD burden remains high due to incomplete adherence to guidelines, difficulties adhering to preventative measures, and the frequency of conditions that increase coronary heart disease risks in patients, including lipid disorders, high blood pressure, and diabetes (Van Camp, 2014). Therefore, clarifying the CVDs’ etiology, pathophysiology, and progression underlying mechanisms and potential therapeutic targets is imperative. The occurrence and progression of CVDs involve multiple cellular processes, in which mitochondria are essential (Dai et al., 2012; Bravo-San Pedro et al., 2017; Tian et al., 2019).

Mitochondria are the powerhouse of cardiac cells (they are the heart unit of cells). Mitochondria are essential during cellular activities such as fatty acid oxidation, oxidative phosphorylation, and energy metabolism. Moreover, mitochondria are involved in adenosine triphosphate (ATP) transfer in the contractile apparatus, Ca^2+^ homeostasis modulation, redox status management, and response to cellular and environmental stress regulation in cardiomyocytes (Pecoraro et al., 2019). CVDs such as cardiac hypertrophy, heart failure, and ischemic cardiomyopathy present abnormalities in the mitochondrial organelle structure and function (mitochondrial damage) (Pecoraro et al., 2019). Proper mitochondrial autophagy facilitates the clearance of damaged mitochondria to promote cardiovascular homeostasis (Campos et al., 2017).

**FIGURE 1:**
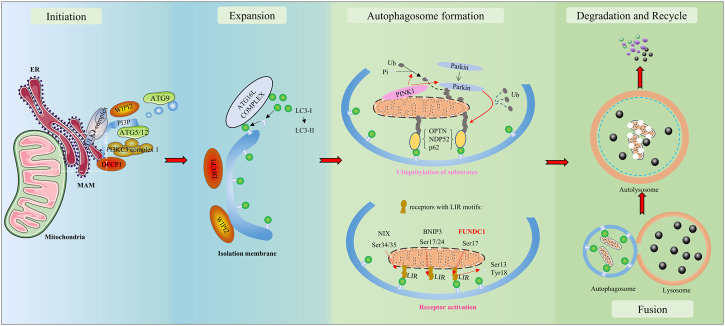
Overview of autophagy/mitophagy. The molecular signals released by damaged mitochondria trigger ubiquitin-mediated and receptor-mediated mitophagy. With the aid of the Unc-51-like kinase 1 (ULK1) complex and class III PI3K (PI3KC3) complex 1, a bilayer lipid membrane enriched in PI3P is formed as part of the endoplasmic reticulum (ER). Then, it could recruit the PI3P effector proteins WD repeat domain phosphoinositide-interacting proteins (WIPIs) and zinc-finger FYVE domain-containing protein 1 (DFCP1), which could attract the autophagy-related protein 8 family (ATG8s), including microtubule-associated protein light chain 3 (LC3) proteins. LC3-I is conjugated to membrane-resident phosphatidylethanolamine (PE) and converted to LC3-II. LC3 has the potential to recognize and engulf labeled proteins and cellular components due to its interaction with LC3-interacting regions (LIRs) of mitophagy receptors. As autophagosomes engulf, they transfer toward and fuse with lysosomes, transforming into autolysosomes. In autolysosomes, the engulfment is degraded and released to the cytosol. Finally, the recycle of nutrients is achieved.

Autophagy is a vital catabolic process with tight regulation under various stresses. As depicted in Figure 1, a bilayer lipid membrane–formed vesicle (the autophagosome) engulfs aged or damaged cellular organelles such as mitochondria, abnormal proteins, or other cellular components and transfers them toward lysosomes. Fused with a lysosome, the autophagosome transforms into an autolysosome. Autolysosomes degrade engulfed materials and release the products to the cytosol, where nutrient recycling occurs. The UNC51-like Ser/Thr kinase (ULK) complex is required during autophagosome formation to initiate autophagy. Cargo receptors with a cargo-binding domain bind the selected materials to microtubule-associated proteins 1A/1B light chain 3 (LC3) *via* the LC3‐interacting region (LIR) to recruit cargo to autophagosomes (Lamb et al., 2013). To summarize briefly, isolation membranes get expanded to form autophagosomes; these get fused to lysosomes to form autolysosomes, and degradation inside the autolysosomes results in unbroken autophagy (i.e., the autophagic flux) (Lamb et al., 2013). An impaired autophagic flux contributes to multiple CVDs, including ischemia/reperfusion (I/R) injury. Failing hearts are known to present a reduced autophagic flux evidenced by the accumulation of autophagy-related markers (Campos et al., 2017). The infarct size in a heart is significantly increased by lysosomal-associated transmembrane protein 4B(LAPTM4B) knockdown-induced impairment of the autophagic flux, but it is reversed upon autophagic flux restoration after overexpression (Gu et al., 2020).

Autophagy of mitochondria, a selective form of autophagy that specifically targets damaged mitochondria, is called mitophagy; it is a mechanism to remove impaired or dysfunctional mitochondria and maintain normal mitochondrial morphology and function in cells. Mitophagy is needed for cells to function well because abundant impaired or dysfunctional mitochondria provide an insufficient supply of energy, overproduce excessive reactive oxygen species (ROS), and activate apoptosis pathways by releasing cytochrome C to the cytoplasm (Wang, 2001; Mishra and Chan, 2016; Vásquez-Trincado et al., 2016; Chan, 2020). Many subcellular organelles, including the endoplasmic reticulum (ER), mitochondria-associated membranes (MAMs), lysosomes, and proteins (FUN14 domain containing 1 [FUNDC1], PTEN-induced putative kinase protein-1 [PINK1]/Parkin, selective autophagy adaptor p62/sequestosome 1 [SQSTM1], and LC3) are involved in mitophagy during CVDs (Tagaya and Arasaki, 2017; Yoo and Jung, 2018). MAMs are regions of the ER that mediate communication between the ER and mitochondria and are the platforms of PINK1/Parkin-dependent mitophagy initiation (Yang et al., 2020). As a MAM-localized protein, FUNDC1 maintains homeostasis of MAMs and plays an essential role in receptor-mediated mitophagy.

FUNDC1 was first reported as a novel hypoxia-induced mitophagy receptor in 2012 (Liu et al., 2012a). It is located on the outer mitochondrial membrane (OMM) with an N-terminal LIR (YEVL) exposed to the cytosol that selectively responds to hypoxia/ischemia stimuli (but not to starvation) (Liu et al., 2012a; Kuang et al., 2016). Various upstream phosphorylases or phosphatases change the phosphorylation states at different FUNDC1 sites to affect the binding affinity of its LIR motif to LC3, thereby promoting or inhibiting mitophagy (Liu et al., 2012a; Feng et al., 2013; Wu et al., 2014a; Chen et al., 2014; Zhou et al., 2018a). In addition, studies have demonstrated that FUNDC1 can tether MAM-specific proteins, facilitate the formation of MAMs, and affect mitochondrial dynamics including the level of Ca^2+^ in the organelle (Wu et al., 2016a; Wu et al., 2016b; Wu et al., 2017). In this review, we collect and summarize the evidence for the roles of FUNDC1 (exclusively on the development of CVDs) describing its interactions with different cellular organelles, its involvement in multiple cellular processes, and its associated signaling pathways.

## 2 FUNDC1-Mediated Mitophagy in CVDs

### 2.1 Mitophagy in CVDs

Studies have demonstrated at least two major mitophagy pathways: ubiquitin-mediated and receptor-mediated mitophagy (Zimmermann and Reichert, 2017). The ubiquitin-mediated mitophagy pathway is mediated by PINK1/Parkin (Eiyama and Okamoto, 2015; Bingol and Sheng, 2016; Nguyen et al., 2016). PINK1 is a molecular sensor of mitochondrial health that constantly surveys the organelle status. In addition, Parkin is an amplifier of mitophagy. Once mitochondria lose their transmembrane potential, PINK1 accumulates at the OMM of impaired or dysfunctional mitochondria and phosphorylates ubiquitin and Parkin at S65. pS65-Ub (the phosphorylated ubiquitin at S65) binds and activates Parkin by destabilizing Parkin’s autoinhibitory interactions and then recruits Parkin from the cytoplasm to the OMM (Nguyen et al., 2016). As E3 ubiquitin ligase, the activated phosphorylated Parkin ubiquitinates various mitochondrial outer-membrane proteins with less specificity. The elongated PINK1 and Parkin proteins form ubiquitin chains that act as molecular signals to further recruit mitophagy receptors, including optineurin (OPTN), nuclear dot protein 52 (NDP52), Tax1-binding protein 1 (TAX1BP1), neighbor of BRCA1 gene 1 (NBR1), and p62, which link ubiquitin chains with LC3 (Dikic and Elazar, 2018). Thus, ubiquitinated proteins in impaired mitochondria can be recognized by cellular mechanisms and get engulfed by autophagosomes to be transferred to lysosomes for degradation.

The known receptor-mediated mitophagy receptors include BCL2 interacting protein 3 such as NIX, also known as (BNIP3L), BCL2 interacting protein 3 (BNIP3), and FUNDC1 in mammalian systems (Liu et al., 2014; Chen et al., 2016). These receptors are integral proteins of the OMM, possessing LIRs, which are the structural basis for LC3 binding to activate mitophagy (Poole and Macleod, 2021). These receptors can be modified by dephosphorylation or phosphorylation under various stresses to affect their affinity for LC3, effectively regulating mitophagy. For instance, BNIP3L-triggered mitophagy can be reversed by PRKA/PKA (protein kinase, AMP-activated)-induced phosphorylation of BNIP3L at Ser212. Activation of the inhibitory phosphorylation site leads to the translocation of BNIP3L from the mitochondria to the cytosol (da Silva Rosa et al., 2021). Phosphorylation of BNIP3 at Ser17/24 sites or NIX at Ser34/35 sites (Rogov et al., 2017) promotes its binding to LC3 and facilitates subsequent mitophagy (Liu et al., 2014). In the case of FUNDC1, post-transcriptional phosphorylation at Ser17 activates mitophagy, while phosphorylation at Ser13 inhibits the process (Wang et al., 2020a). Thus, mitophagy mediated by these two pathways contributes to the clearance of damaged mitochondria and might be mutually affected.

Proper mitophagy guarantees homeostasis of mitochondria in cells and exerts protective effects on the cardiovascular system, while insufficient or excessive mitophagy may be detrimental. Atherosclerosis, hypertension, ischemia/reperfusion injury, myocardial infarction, cardiac hypertrophy, heart failure, and metabolic cardiomyopathy consistently exhibit mitophagy-involved pathological processes. *In vivo* experiments have shown that knockout of pivotal mitophagy molecules can affect the phenotype and severity of diseases. For example, deletion of PINK1 leads to more severe cardiac hypertrophy and left ventricle dysfunction in mice than those in wild-type and heterozygous mice (Billia et al., 2011), while Parkin-knockout mice are vulnerable to myocardial infarction induced by ligation of the proximal left anterior descending coronary artery and present a low survival rate (Kubli et al., 2013). Similarly, the mammalian target of rapamycin complex 1 (mTORC1) activation in dietary protein-driven atherosclerotic plaques inhibits mitophagy (its downstream effect) and results in a buildup of dysfunctional mitochondria that contribute to a rise in plaque complexity (Zhang et al., 2020). Likewise, the NIX expression has been found to be decreased in human atherosclerosis. Silencing the NIX expression in murine macrophage cells reduced NIX-mediated mitophagy, enhanced oxidized low-density lipoprotein (ox-LDL)-induced macrophage pyroptosis, and led to formation of unstable plaques (Peng et al., 2020). Therefore, numerous chemicals targeting the modulation of mitophagy may alleviate or exacerbate different cardiovascular dysfunctions (Hsu et al., 2015; Ma et al., 2018; Qiao et al., 2018; Yang et al., 2021).

Cardiomyocytes, cardiac fibroblasts (CFs), endothelial cells (ECs), vascular smooth muscle cells (VMSCs), macrophages, and other cell types need to work in an organized manner to keep the cardiovascular system functioning well and maintain a low disease risk. Improper mitophagy can alter the functions of cells and result in the occurrence and progression of diseases. Inhibited mitophagy aggravates lipid accumulation and leads to heart dysfunction (Tong et al., 2019). A mitophagy imbalance renders cardiomyocytes apoptotic under I/R stress (Li et al., 2019a). PINK1/Parkin-mediated mitophagy is upregulated in endothelial cells under metabolic stress to protect mitochondrial integrity and prevent metabolic stress–induced endothelial injury (Wu et al., 2015). The melatonin-induced suppression of mitophagy protects microvascular endothelial cells against I/R injury (Zhou et al., 2017a). In addition, inhibited mitophagy suppresses activation of cardiac fibroblasts but promotes apoptosis (Gao et al., 2020a), while enhanced mitophagy restrains proliferation and apoptosis of VMSCs (Swiader et al., 2016; Chen et al., 2020). Thus, mitophagy in various cell types contributes to cardiac function.

### 2.2 Structure and Post-transcriptional Modification of FUNDC1 for Mitophagy

FUNDC1 is a protein with three *α*-helix transmembrane domains at the OMM and characteristics similar to those of other mitophagy receptors. Its LIR motif (Y18-E19-V20-L21 at the N-terminal region of FUNDC1 in the cytoplasm) has the classic tetrapeptide W/F/YxxL/I sequence for interaction with LC3, which links it to the ATG5-dependent core autophagic machinery (Liu et al., 2012a; Kuang et al., 2016). Mutants of Y18A, V20A, and L21A or complete deletion of the LIR sequence display a reduced or even abolished affinity of FUNDC1 for LC3 binding that disrupts mitophagy; other FUNDC1 mutations have no effects (Liu et al., 2012a; Kuang et al., 2016). Phosphorylation is the main post-transcriptional modification of FUNDC1 that regulates mitophagy. Three key residues of FUNDC1, Ser13, Ser17, and Tyr18, get phosphorylated and modify the binding affinity of FUNDC1 for LC3 and consequently influence mitophagy (Lv et al., 2017).

**FIGURE 2:**
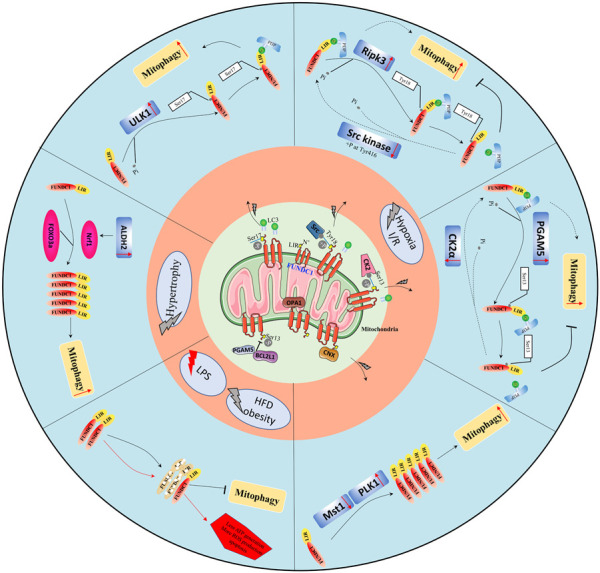
Overview of the protective role of FUNDC1-mediated mitophagy in cardiovascular diseases. Physiologically, FUNDC1 phosphorylation at Ser13 and Tyr18 or dephosphorylation at Ser17; OPA1-FUNDC1 connection, FUNDC1-calnexin association, and the BCL2L1-PGAM5 complex making FUNDC1 dormant. Under abnormal conditions, FUNDC1 could be dephosphorylated at Ser13, Tyr18, and or phosphorylated at Ser17 by various protein kinases such as Src kinase, ULK1, PGAM5, and others remaining to be identified to increase its affinity with LC3 to promote mitophagy.

Under normal conditions, as depicted in Figure 2, FUNDC1 phosphorylation at Ser13 (Chen et al., 2014; Zhou et al., 2018a) and Tyr-18 (Feng et al., 2013; Zhou et al., 2017b) or dephosphorylation at Ser17 (Wu et al., 2014a) both decrease the affinity of FUNDC1 for LC3 by altering its stereochemical properties. The phosphorylation of FUNDC1 at Ser13 and Tyr18 is mediated by casein kinase 2 (CK2) kinases and Src kinase, respectively (Chen et al., 2014). The affinity of the dephosphorylated FUNDC1 peptide (at Ser17) for LC3 (at Lys49) is ∼3-fold weaker than that of the phosphorylated FUNDC1 peptide (Lv et al., 2017). The direct optic atrophy 1 (OPA1)-FUNDC1 connection and the FUNDC1-calnexin association block other FUNDC1 interactions, and the BCL2L1 (BH3 domain)–PGAM5L (a member of the phosphoglycerate mutase family) complex inhibits FUNDC1 dephosphorylation at Ser13; all these molecular interactions inhibit FUNDC1 activities (Wu et al., 2014b). Additionally, the membrane-associated RING-CH protein 5 (MARCH5), a mitochondrial E3 ligase, mediates FUNDC1 ubiquitylation and degradation by directly interacting with it at lysine 119. Thus, FUNDC1 is inactivated in healthy hearts. Moderately inactivated FUNDC1 may interact with the F-box protein FBXL2 to maintain the mitochondrial integrity or it may form a complex with heat shock protein 70 (HSC70) to promote the mitochondrial translocation of unfolded cytosolic proteins and maintain the cardiac function (Li et al., 2019b; Ren et al., 2020).

Under abnormal conditions like hypoxia, two pathways regulate FUNDC1 to mediate mitophagy: a kinase-mediated pathway and an interactional protein-regulated pathway. Tyr416-phosphorylated Src enhances Tyr18 dephosphorylation of FUNDC1, increasing its binding ability for LC3 and promoting mitophagy (Feng et al., 2013; Zhou et al., 2017b). Similarly, CK2 dissociates from FUNDC1 and allows PGAM5 to dephosphorylate it at Ser13, strengthening its interaction with LC3 (Chen et al., 2014; Zhou et al., 2018a). ULK1 translocates to mitochondria and activates FUNDC1 by phosphorylating its Ser17, and ULK1 deletion inhibits FUNDC1 activation (Wang et al., 2020a). Under stress, phosphorylated FUNDC1 dissociates from OPA1 and interacts instead with dynamin-related protein 1 (DRP1) to enhance mitochondrial fission and mitophagy (Wu et al., 2016b). During mitophagy under hypoxic conditions, the cytosolic loop of FUNDC1 is exposed (due to an attenuated FUNDC1/calnexin association) and interacts with DRP1 (Wu et al., 2016b). Disorders of mitochondrial dynamics, including imbalanced fission and fusion, are prerequisites for mitophagy. Hypoxia induces BCL2L1 degradation, increases PGAM5 dissociation, and then enhances dephosphorylation of FUNDC1 at Ser13 (Ma et al., 2020). Additionally, the decreased endogenous MARCH5 expression significantly inhibits FUNDC1 degradation and promotes mitophagy (Chen et al., 2017). Other regulators of FUNDC1 have been identified: the Nod‐like receptor X1 (NLRX1), in mitochondria, negatively regulates phosphorylated Tyr18 FUNDC1 levels (Li et al., 2021), and the LncRNA MEG3 overexpression induces dephosphorylation of FUNDC1 at Tyr18 by interacting with the 3′UTR of Rac1 to inhibit its expression (Wang et al., 2021a). The transcriptional and post-transcriptional controls of FUNDC1 have also been shown to be important. Under normoxic conditions, the negative regulatory factor microRNA-137 is constitutively expressed; it targets the 3′ UTR of the FUNDC1 mRNA suppressing its translation to attenuate FUNDC1-LC3 associations. Under hypoxic conditions, the microRNA-137 is downregulated. At the transcriptional level, mitophagy is enhanced by the peroxisome proliferator-activated receptor gamma coactivator 1-alpha (PGC-1a) protein, which induces the FUNDC1 expression by upregulating the nuclear respiratory factor 1 (NRF1) expression, a factor that binds FUNDC1 at -186/-176 sites (Liu et al., 2021a).

In summary, FUNDC1 is crucial to receptor-mediated mitophagy. Proper mitophagy helps restore cardiac function after hypoxia, I/R, and other stresses. However, a review of FUNDC1-mediated mitophagy in CVDs has not been published.

### 2.3 FUNDC1-Mediated Mitophagy in CVDs

#### 2.3.1 Hypoxia and I/R

Cardiomyocytes need to generate ATP through oxidative phosphorylation in the respiratory chain of mitochondria, which is highly dependent on oxygen consumption. When hypoxia or ischemia occurs, mitochondria are the first organelles to exhibit extensive fission, loss of membrane potential, and release of proapoptotic signals that eventually led to cell death. I/R injury, which is accompanied by the mitochondrial Ca^2+^ overload, ROS generation, autophagy failure, platelet activation, and microthrombosis (Aghaei et al., 2019; Yang et al., 2019), is a common clinical condition due to rapid revascularization treatments after acute myocardial infarction. Revascularization brings oxygen and nutrition to “suffocated cardiomyocytes,” but it simultaneously promotes cell death (Li et al., 2019a; Wang et al., 2020b). Mitophagy plays a protective role in I/R injury. Under ischemia, mitophagy is thought to be cardioprotective due to its removal of impaired mitochondria, reduction of mitochondrial ROS (mROS) and apoptosis, and reduced inflammation (Yang et al., 2019; Xin and Lu, 2020; Yu et al., 2020). OPA1-induced mitophagy and FUNDC1-dependent mitophagy could offer cardioprotection against ischemia (Li et al., 2018; Xin and Lu, 2020), while the knockout of Parkin causes extensive cardiac injury due to mitochondrial dysfunction and mitophagy inhibition (Kubli et al., 2013). Most other studies have also suggested a cardioprotective role for mitophagy during ischemia. However, mitophagy may exert detrimental effects during the reperfusion phase. According to a published hypothesis, fragmented mitochondria and excessive mitophagy could reduce the necessary ATP supply, leading to cell death (Anzell et al., 2018; Yang et al., 2019). Thus, proper mitophagy guarantees the homeostasis of mitochondria to maintain a normal cellular physiology, but mitophagy dysregulation is pathogenic and even fatal for cells.

Studies have demonstrated that FUNDC1 plays an essential role in mitophagy under hypoxia or I/R conditions. Li et al. reported that the ULK1 signaling pathway mediates FUNDC1 phosphorylation, leading to increased mitophagy levels and cardiac function protection under ischemia (Li et al., 2018). Zhou et al. demonstrated that FUNDC1-mediated mitophagy gets activated to prevent myocardial apoptosis during ischemia, while upregulation of Ripk3 can phosphorylate Tyr18 in FUNDC1 during reperfusion to inhibit FUNDC1-dependent mitophagy and increase necrosis (Zhou et al., 2017b). Zhang et al. found a dual role for mitophagy in platelets, where FUNDC1–knocked-out platelets presented reduced but sustained mitophagy activity and caused more injuries during the late stages of I/R in the heart (Zhang et al., 2016). The same researchers also generated a cell-penetrating peptide to block mitophagy *in vivo* by intraperitoneal administration to prevent mitochondrial dysfunctions and platelet inactivation, which could become a new strategy potentially applicable in the clinical setting. Zhou et al. studied the association between mitophagy and microvascular permeability and found that under I/R stress, the upregulated nuclear receptor subfamily 4 group A member 1 (NR4A1) induces CK2α to phosphorylate the mitochondrial fission factor (Mff) and FUNDC1, thereby enhancing mitochondrial fission and inhibiting mitophagy, resulting in microvascular hyperpermeability, endothelial cell apoptosis, and damage (Zhou et al., 2018b). Genetic deletion of CK2α was also proved by Zhou et al. to protect cardiomyocytes from I/R injury *via* decreased Ser13 phosphorylation of FUNDC1 to promote mitophagy and to prevent mitochondrial damage and apoptosis (Zhou et al., 2018a). In addition, some kinases, such as mammalian STE20-like kinase 1 (Mst1) and polo-like kinase 1 (PLK1), have also been associated with FUNDC1-mediated mitophagy *in vivo* and *in vitro* under I/R stimuli (Yu et al., 2019; Mao et al., 2021).

Interestingly, mitophagy contributes to functional changes in different organs. For example, electroacupuncture preconditioning has a protective effect in patients undergoing heart valve replacement surgery, and this is caused by inhibition of mitophagy mediated by the mTORC1-ULK1-FUNDC1 pathway (Xiao et al., 2020). The protective effect of electroacupuncture pretreatment on cerebral I/R injury also correlates with p-mTORC1 mitophagy (Mao et al., 2020). Moreover, the transient receptor potential cation channel subfamily V member 1 (TRPV1) factor alleviates I/R-induced acute renal injury (Wei et al., 2020). The TRPV1-mediated transient Ca^2+^ influx activates AMP-activated protein kinase (AMPK) and reduces FUNDC1 transcription. This indicates that the Ca^2+^ influx and mitophagy are both regulators during I/R. In addition, FUNDC1-mediated mitophagy (triggered by the activated phosphorylation of AMPK) contributes to the protective effect of the tissue-type plasminogen activator during cerebral I/R injury (Cai et al., 2021). Mitophagy is also considered a key mechanism during intestinal I/R injury. Downregulated NLRX1 promotes phosphorylation of FUNDC1 in intestinal I/R injury (Li et al., 2021). Phosphorylated FUNDC1 decouples from the nitrophenylphosphatase domain and non‐neuronal SNAP25‐like protein homologs 1 and 2 (NIPSNAP 1 and 2; mitophagy signaling proteins on the outer membrane of damaged mitochondria) and then fails to trigger mitophagy (Li et al., 2021). Similarly, during acute kidney injury, ischemia preconditioning activates FUNDC1 mitophagy (through post-transcriptional phosphorylation at Ser17) to mitigate I/R injury-mediated renal injury (Wang et al., 2020a). Downregulated FUNDC-1, the *C. elegans* ortholog of FUNDC1, protects the worm against injury in a model of hypoxia-reoxygenation stress. This protection depends on activation of the transcription factor associated with stress-1 (ATFS-1), the central transcription factor that regulates the mitochondrial unfolded protein response (Lim et al., 2021). Taken together, most evidence points to FUNDC1-mediated mitophagy being essential against hypoxia and I/R injury; FUNDC1 may be a promising therapeutic target.

#### 2.3.2 Cardiac Hypertrophy and Remodeling

Cardiac hypertrophy is a manifestation of enlargement of individual cardiomyocytes (not an increase in the number of cells) due to various pathological stresses, such as pressure overload, infarction, metabolic disturbances, or structural heart disease, eventually developing into heart failure. Pathological cardiac hypertrophy involves changes in multiple cellular processes, including excessive protein synthesis and inhibition of selective autophagy (Nakamura and Sadoshima, 2018; Zhou et al., 2020). Studies have demonstrated that activated mitophagy can mitigate cardiac hypertrophy. Lysocardiolipin acyltransferase 1 (ALCAT1) deletion upregulates PINK1 and mitigates oxidative stress, insulin resistance, and mitochondrial dysfunction *via* activation of PINK1-mediated mitophagy and alleviating cardiac hypertrophy (Liu et al., 2012b). Macrophage migration inhibitory factor (MIF) depletion hinders the activation of Parkin-dependent mitophagy by regulating AMPK-mTOR signaling pathways to exacerbate the hypertrophy induced by pressure overload (Xu et al., 2014). In addition, PINK1 autophosphorylation can also recruit Parkin to initiate mitophagy, exerting a protective effect on angiotensin II (ANG II)-induced hypertrophy (Xiong et al., 2018).

FUNDC1-dependent mitophagy has also been shown to play a critical role in cardiac hypertrophy. In a mouse model of cardiac hypertrophy induced by continuous administration of isoproterenol (ISO), Liu et al. reported autophagy inhibition as the LC3II/LC3I ratio decreased, and the FUNDC1 expression was downregulated. This was confirmed by *in vitro* experiments using neonatal rat cardiomyocytes (NRCMs), in which the hypertrophy could be alleviated by baicalein, a flavonoid extracted from the root of *Scutellaria baicalensis* (Liu et al., 2021b). Mechanistically, baicalein binds directly to FOXO3a (a transcription factor) and transactivates FUNDC1. In another study by Li et al, FUNDC1-related mitophagy was associated with cardiac hypertrophy in a mouse model of transaortic constriction (TAC) and an *in vitro* model of NRCMs induced using ANG II (Li et al., 2020). ALDH2 activated by alpha-lipoic acid (α-LA), a well-known antioxidant, governs the activation of Nrf1-FUNDC1. Nrf1, a member of the Cap-N-Collar family of regulatory proteins, binds to the 5′ promoter of FUNDC1 to modulate the FUNDC1 expression directly. Thus, the evidence indicates that FUNDC1-mediated mitophagy is involved in cardiac hypertrophy and that interference with the associated signaling pathways could prevent the progression or deterioration of the disease.

#### 2.3.3 FUNDC1-Mediated Mitophagy in Obesity- or High-Fat Diet Intake–Induced Heart Dysfunction

Obesity coexists with reduced autophagy and mitophagy, alongside the inflammation, oxidative stress, lipotoxicity, and apoptosis, (Lavallard et al., 2012; Guo et al., 2013; Zhang et al., 2018; Shao et al., 2020) that together may lead to heart dysfunction. Mitophagy markers such as Parkin and BNIP3 are downregulated following HFD feeding (Zeinvand-Lorestani et al., 2018; Thomas et al., 2019). However, a study reported that HFD feeding in mice consistently activates mitophagy, as evaluated with Mito-Keima (Tong et al., 2019). These researchers also found that inhibition of mitophagy by deletion of ATG7 or Parkin in an HFD-induced mouse model can increase lipid accumulation and worsen heart dysfunction, while activation of mitophagy by TB1 (Tat-Beclin1) injection exerts the opposite effect (Tong et al., 2019).

Wu et al. found that impaired mitophagy and compromised mitochondrial quality control due to FUNDC1 knockout lead to obesity and insulin resistance in mice *via* the MAPK/JUN pathway and the inflammatory response (Wu et al., 2019a). In addition, Ren et al. found that FUNDC1 and mitophagy were downregulated in a HFD-induced mouse model and that FUNDC1-knockout mice were more vulnerable to HFD-induced cardiac hypertrophy, fibrosis, and insufficiency, *via* interaction with FBXL2 in an inositol 1,4,5-trisphosphate receptor type 3 (IP3R3)-dependent manner (Ren et al., 2020). Their study also confirmed that loss of FUNDC1-mediated mitophagy and increased fatty acid synthase acyl-CoA synthetase long-chain 4 (ACSL4)-mediated ferroptosis led to cardiac remodeling and contractile anomaly in FUNDC1-knockout mice under an HFD-induced model (Pei et al., 2021). However, Fu et al. found that skeletal muscle–specific FUNDC1-knockout mice present impaired mitochondrial energetics in the skeletal muscle and exercise performance, but the mice are markedly resistant to HFD-induced obesity with high systemic insulin sensitivity and glucose tolerance (Fu et al., 2018). The mechanism might be that FUNDC1 deficiency upregulated the expression of fibroblast growth factor 21(FGF21), a peptide hormone that regulates energy homeostasis. Based on the results of these studies, FUNDC1-related mitophagy regulates cardiac metabolism under obesity or HFD stress and may be a potential target to prevent obesity-associated cardiac injury. However, underlying mechanisms remain to be clarified.

## 3 Other Roles of FUNDC1 at MAMs Affecting Heart Dysfunction

MAMs are the sites connecting mitochondria and the ER through protein–protein or protein–lipid complex tethers, at which these two subcellular organelles exchange contents and execute fundamental biological processes jointly (Ca^2+^ and lipid exchange, inflammation, and oxidative stress) (Wu and Zou, 2019; Gao et al., 2020b; Silva-Palacios et al., 2020). Emerging evidence has indicated the importance of MAM in CVDs. FUNDC1 is a MAM-related protein important for MAM formation, Ca^2+^ exchange between the ER and mitochondria, and mitochondrial morphology (Wu et al., 2016b; Wu et al., 2017; Wu et al., 2019b).

### 3.1 FUNDC1 and MAM Formation

FUNDC1 has been found enriched at MAMs under stress and to facilitate ER and mitochondrial tethering by interacting with ER proteins such as calnexin and IP3R2 (ER-resided inositol 1,4,5-trisphosphate type 2 receptor) (Wu et al., 2016b; Wu et al., 2017; Wu et al., 2019b). Under hypoxia, FUNDC1 accumulates at the MAM and exhibits a dynamic interaction with the MAM-related protein calnexin (Wu et al., 2016b). In cardiomyocyte-specific FUNDC1-knockout mice, the connection between the ER and mitochondria in cardiomyocytes is disrupted, and the mice present few MAMs and MAM-related proteins (IP3R2 and PACS-2 [phosphofurin acidic cluster sorting protein 2]), a picture consistent with that in the H9C2 cell line (Wu et al., 2017). In a high glucose-induced *in vitro* model, the FUNDC1 overexpression promoted MAM formation, and FUNDC1 ablation inhibited it (Wu et al., 2019b). Wang et al. found similar phenotypes in FUNDC1-deleted endothelial cells (EC) and EC-specific FUNDC1-knockout mice (Wang et al., 2021b). Based on the evidence, FUNDC1 is a MAM-related protein that participates in the formation and function of MAMs.

### 3.2 FUNDC1 and Calcium Homeostasis

During the cardiac cycle, Ca^2+^ is rapidly released to the cytosol from the sarcoplasmic reticulum (SR) and then restored (Gambardella et al., 2018). Appropriate calcium handling is vital for excitation–contraction (EC) coupling of cardiomyocytes, and calcium flux disruption eventually leads to heart dysfunction. Mitochondria can act as Ca^2+^ buffers, and they are also involved in Ca^2+^ reuptake, but Ca^2+^ overload in mitochondria can be harmful and cause heart failure (Brookes et al., 2004; Gambardella et al., 2018). The FUNDC1-mediated MAM is an important structure that regulates intracellular calcium homeostasis. Specific FUNDC1-knockout cardiomyocytes present decreased cytoplasmic and mitochondrial Ca^2+^ and increased ER Ca^2+^, while FUNDC1-overexpressing cardiomyocytes display the opposite effects (which can be abolished by silencing IP3R2) (Wu et al., 2017). Ablation of FUNDC1 decreases mitochondrial Ca^2+^ (*via* MAMs induced by high glucose) and also inhibits ROS production and cell apoptosis, preventing cardiac dysfunction *in vivo* (Wu et al., 2019b).

### 3.3 FUNDC1 and Mitochondrial Dynamics

Studies have reported that deletion of FUNDC1 results in elongated mitochondria in cardiomyocytes (Wu et al., 2016b; Chen et al., 2016; Wu et al., 2017). Wu et al. observed both fewer absolute fission events and a decreased ratio of fission to fission and fusion events in specific FUNDC1-knockout cardiomyocytes *via* time-lapse confocal imaging (Wu et al., 2017). They also found that FUNDC1 loss inhibits the integrity of MAMs, causing increased mitochondrial and intracellular Ca^2+^ concentrations and leading to cardiac dysfunction (Wu et al., 2017). As mentioned, FUNDC1 coordinates mitochondrial dynamics and mitophagy at MAMs by interacting with DRP1 and OPA1. FUNDC1 ablation suppresses the mitochondrial fission 1 protein (Fis1) expression by reducing the binding of the cAMP response element-binding protein (CREB) in the Fis1 promoter and inhibiting mitochondrial fission in cardiomyocytes (Wu et al., 2017). In HeLa cells under hypoxia, FUNDC1 is involved in mitochondrial fission *via* its Mff interaction (Wu et al., 2016b). In addition, USP19 (an ER-resident deubiquitinase) can bind FUNDC1 and deubiquitinate it at the MAMs leading to DRP1 oligomerization and promotion of mitochondrial division (Chai et al., 2021).

## 4 FUNDC1 Regulates the Production of ROS and Apoptosis in CVDs

Impaired mitochondria with an altered calcium buffering system generate less ATP and more ROS, eventually leading to mitochondria-related cell apoptosis. Studies have established an association between FUNDC1 and ROS generation and apoptosis (Zhang et al., 2016; Wu et al., 2017; Wu et al., 2019b; Huang et al., 2020; Jiang et al., 2021). Huang et al. found that ablation of FUNDC1 enhances the production of ROS and interleukin 1-β (IL1-β) in macrophages treated with combined lipopolysaccharide (LPS) and nigericin *in vivo* and *in vitro* through the regulation of mitophagy, while the overexpression of FUNDC1 (but not of its Y18A/L21A mutant) can reverse this effect *in vitro* (Huang et al., 2020). In the H9C2 model of septic cardiomyopathy, Jiang et al. found that the ROS generation and apoptosis related to FUNDC1-mediated mitophagy can be attenuated and inhibited by irisin (Jiang et al., 2021). Wang et al. observed similar results in the AC16 human ventricular cardiomyocyte cell line incubated with LPS (Wang et al., 2021c). Wu et al. also confirmed that simple FUNDC1 deletion is sufficient to promote cardiomyocyte apoptosis and heart failure *in vivo* in cardiomyocyte-specific FUNDC1-knockout mice (Wu et al., 2017). However, it is interesting to note that FUNDC1 knockout in Akita mice inhibits excessive ROS production and improves the mitochondrial membrane potential in diabetic hearts compared with the effects in non–FUNDC1-knockout Akita mice (Wu et al., 2019b).

## 5 Conclusion

FUNDC1 (a novel identified receptor of mitophagy at the MAM) plays an important role in mitochondrial homeostasis, MAM-related cellular processes, and mitochondria-mediated apoptosis. We collected evidence demonstrating that FUNDC1 is closely involved with various CVDs. Activated FUNDC1-mediated mitophagy has been proposed to play protective roles in I/R injury, cardiac hypertrophy, and obesity-induced cardiomyopathy. FUNDC1-mediated mitophagy may be stabilized by phosphorylation/dephosphorylation of the three key residues of FUNDC1: Ser13, Ser17, and Tyr18. Thus, these sites are promising therapeutic targets to exploit small molecule drugs that can induce protective mitophagy. This is an enormous challenge that needs to be further explored.

Abundant impaired mitochondria generate high levels of ROS and induce apoptosis, two phenomena that are also affected by FUNDC1. The interaction of FUNDC1 and MAM-located proteins regulates mitochondrial morphology and calcium homeostasis in the cytosol and mitochondria, ensuring cardiac contractility and normal heart function. Good quality and detailed studies indicate that FUNDC1 and its associated cellular pathways may be a promising therapeutic target for the prevention and management of CVDs. However, the association between FUNDC1 and mROS in CVDs needs clarification.

Some important roles of FUNDC1 in CVDs have been revealed by laboratory experiments, but gaps remain that hamper our understanding of the complex pathophysiological processes at play; more studies are needed before turning laboratory results into effective and safe translational medicine. Many interventional approaches used in the laboratory are not currently available in clinical settings. However, some studies have made excellent attempts at demonstrating their utility. Cell-permeable functional peptides composed of the HIV-1 Tat protein transduction domain have been proven effective to induce FUNDC1-mediated mitophagy activity in cell tests. Similarly, intraperitoneal injection of well-designed synthetic cell-penetrating peptides *in vivo* could lead to satisfactory manipulation of FUNDC1-mediated mitophagy. Unfortunately, in contrast to the many kinases involved in FUNDC1-mediated mitophagy processes tested, no inhibitors or agonists of those corresponding kinases have been studied *in vivo*. More investigations and innovations are needed before treatments targeting this molecule can be applied in clinical settings.

## References

[B1] Aghaei M. Motallebnezhad M. Ghorghanlu S. Jabbari A. Enayati A. Rajaei M. (2019). Targeting Autophagy in Cardiac Ischemia/reperfusion Injury: A Novel Therapeutic Strategy. J. Cel Physiol 234, 16768–16778. 10.1002/jcp.28345 30807647

[B2] Anzell A. R. Maizy R. Przyklenk K. Sanderson T. H. (2018). Mitochondrial Quality Control and Disease: Insights into Ischemia-Reperfusion Injury. Mol. Neurobiol. 55, 2547–2564. 10.1007/s12035-017-0503-9 28401475 PMC5636654

[B3] Billia F. Hauck L. Konecny F. Rao V. Shen J. Mak T. W. (2011). PTEN-inducible Kinase 1 (PINK1)/Park6 Is Indispensable for normal Heart Function. Proc. Natl. Acad. Sci. 108, 9572–9577. 10.1073/pnas.1106291108 21606348 PMC3111326

[B4] Bingol B. Sheng M. (2016). Mechanisms of Mitophagy: PINK1, Parkin, USP30 and beyond. Free Radic. Biol. Med. 100, 210–222. 10.1016/j.freeradbiomed.2016.04.015 27094585

[B5] Bravo-San Pedro J. M. Kroemer G. Galluzzi L. (2017). Autophagy and Mitophagy in Cardiovascular Disease. Circ. Res. 120, 1812–1824. 10.1161/circresaha.117.311082 28546358

[B6] Brookes P. S. Yoon Y. Robotham J. L. Anders M. W. Sheu S.-S. (2004). Calcium, ATP, and ROS: a Mitochondrial Love-Hate triangle. Am. J. Physiology-Cell Physiol. 287, C817–C833. 10.1152/ajpcell.00139.2004 15355853

[B7] Cai Y. Yang E. Yao X. Zhang X. Wang Q. Wang Y. (2021). FUNDC1-dependent Mitophagy Induced by tPA Protects Neurons against Cerebral Ischemia-Reperfusion Injury. Redox Biol. 38, 101792. 10.1016/j.redox.2020.101792 33212415 PMC7679257

[B8] Campos J. C. Queliconi B. B. Bozi L. H. M. Bechara L. R. G. Dourado P. M. M. Andres A. M. (2017). Exercise Reestablishes Autophagic Flux and Mitochondrial Quality Control in Heart Failure. Autophagy 13, 1304–1317. 10.1080/15548627.2017.1325062 28598232 PMC5584854

[B9] Chai P. Cheng Y. Hou C. Yin L. Zhang D. Hu Y. (2021). USP19 Promotes Hypoxia-Induced Mitochondrial Division via FUNDC1 at ER-Mitochondria Contact Sites. J. Cel Biol 220. 10.1083/jcb.202010006 PMC8127008 33978709

[B10] Chan D. C. (2020). Mitochondrial Dynamics and its Involvement in Disease. Annu. Rev. Pathol. Mech. Dis. 15, 235–259. 10.1146/annurev-pathmechdis-012419-032711 31585519

[B11] Chen G. Han Z. Feng D. Chen Y. Chen L. Wu H. (2014). A Regulatory Signaling Loop Comprising the PGAM5 Phosphatase and CK2 Controls Receptor-Mediated Mitophagy. Mol. Cel 54, 362–377. 10.1016/j.molcel.2014.02.034 24746696

[B12] Chen M. Chen Z. Wang Y. Tan Z. Zhu C. Li Y. (2016). Mitophagy Receptor FUNDC1 Regulates Mitochondrial Dynamics and Mitophagy. Autophagy 12, 689–702. 10.1080/15548627.2016.1151580 27050458 PMC4836026

[B13] Chen Y. Li S. Guo Y. Yu H. Bao Y. Xin X. (2020). Astaxanthin Attenuates Hypertensive Vascular Remodeling by Protecting Vascular Smooth Muscle Cells from Oxidative Stress-Induced Mitochondrial Dysfunction[J]. Oxid Med. Cel Longev 2020, 4629189. 10.1155/2020/4629189 PMC7178508 32351673

[B14] Chen Z. Liu L. Cheng Q. Li Y. Wu H. Zhang W. (2017). Mitochondrial E3 Ligase MARCH 5 Regulates FUNDC 1 to fine‐tune Hypoxic Mitophagy. EMBO Rep. 18, 495–509. 10.15252/embr.201643309 28104734 PMC5331199

[B15] da Silva Rosa S. C. Martens M. D. Field J. T. Nguyen L. Kereliuk S. M. Hai Y. (2021). BNIP3L/Nix-induced Mitochondrial Fission, Mitophagy, and Impaired Myocyte Glucose Uptake Are Abrogated by PRKA/PKA Phosphorylation. Autophagy 17, 2257–2272. 10.1080/15548627.2020.1821548 33044904 PMC8496715

[B16] Dai D.-F. Rabinovitch P. S. Ungvari Z. (2012). Mitochondria and Cardiovascular Aging. Circ. Res. 110, 1109–1124. 10.1161/circresaha.111.246140 22499901 PMC3867977

[B17] Dikic I. Elazar Z. (2018). Mechanism and Medical Implications of Mammalian Autophagy. Nat. Rev. Mol. Cel Biol 19, 349–364. 10.1038/s41580-018-0003-4 29618831

[B18] Eiyama A. Okamoto K. (2015). PINK1/Parkin-mediated Mitophagy in Mammalian Cells. Curr. Opin. Cel Biol. 33, 95–101. 10.1016/j.ceb.2015.01.002 25697963

[B19] Feng D. Liu L. Zhu Y. Chen Q. (2013). Molecular Signaling toward Mitophagy and its Physiological Significance. Exp. Cel Res. 319, 1697–1705. 10.1016/j.yexcr.2013.03.034 23603281

[B20] Fu T. Xu Z. Liu L. Guo Q. Wu H. Liang X. (2018). Mitophagy Directs Muscle-Adipose Crosstalk to Alleviate Dietary Obesity. Cel Rep. 23, 1357–1372. 10.1016/j.celrep.2018.03.127 29719250

[B21] Gambardella J. Trimarco B. Iaccarino G. Santulli G. (2018). New Insights in Cardiac Calcium Handling and Excitation-Contraction Coupling. Adv. Exp. Med. Biol. 1067, 373–385. 10.1007/5584_2017_106 28956314 PMC5889357

[B22] Gao P. Yan Z. Zhu Z. (2020). Mitochondria-Associated Endoplasmic Reticulum Membranes in Cardiovascular Diseases. Front. Cel Dev. Biol. 8, 604240. 10.3389/fcell.2020.604240 PMC7680862 33240899

[B23] Gao Q. Y. Zhang H. F. Tao J. Chen Z. T. Liu C. Y. Liu W. H. (2020). Mitochondrial Fission and Mitophagy Reciprocally Orchestrate Cardiac Fibroblasts Activation. Front Cel Dev Biol 8, 629397. 10.3389/fcell.2020.629397 PMC7874126 33585469

[B24] Gu S. Tan J. Li Q. Liu S. Ma J. Zheng Y. (2020). Downregulation of LAPTM4B Contributes to the Impairment of the Autophagic Flux via Unopposed Activation of mTORC1 Signaling during Myocardial Ischemia/Reperfusion Injury. Circ. Res. 127, e148–e165. 10.1161/CIRCRESAHA.119.316388 32693673

[B25] Guo R. Zhang Y. Turdi S. Ren J. (2013). Adiponectin Knockout Accentuates High Fat Diet-Induced Obesity and Cardiac Dysfunction: Role of Autophagy. Biochim. Biophys. Acta (Bba) - Mol. Basis Dis. 1832, 1136–1148. 10.1016/j.bbadis.2013.03.013 PMC3796200 23524376

[B26] Hsu P. Liu X. Zhang J. Wang H.-G. Ye J.-M. Shi Y. (2015). Cardiolipin Remodeling by TAZ/tafazzin Is Selectively Required for the Initiation of Mitophagy. Autophagy 11, 643–652. 10.1080/15548627.2015.1023984 25919711 PMC4502692

[B27] Huang J. Zhu T. Rong R. You M. Ji D. Li H. (2020). FUN14 Domain‐containing 1-mediated Mitophagy Suppresses Interleukin-1β Production in Macrophages. Int. Immunopharmacology 88, 106964. 10.1016/j.intimp.2020.106964 33182075

[B28] Jiang X. Cai S. Jin Y. Wu F. He J. Wu X. (2021). Irisin Attenuates Oxidative Stress, Mitochondrial Dysfunction, and Apoptosis in the H9C2 Cellular Model of Septic Cardiomyopathy through Augmenting Fundc1-dependent Mitophagy. Oxid Med. Cel Longev 2021, 2989974. 10.1155/2021/2989974 PMC8390168 34457111

[B29] Kuang Y. Ma K. Zhou C. Ding P. Zhu Y. Chen Q. (2016). Structural Basis for the Phosphorylation of FUNDC1 LIR as a Molecular Switch of Mitophagy. Autophagy 12, 2363–2373. 10.1080/15548627.2016.1238552 27653272 PMC5173264

[B30] Kubli D. A. Zhang X. Lee Y. Hanna R. A. Quinsay M. N. Nguyen C. K. (2013). Parkin Protein Deficiency Exacerbates Cardiac Injury and Reduces Survival Following Myocardial Infarction. J. Biol. Chem. 288, 915–926. 10.1074/jbc.m112.411363 23152496 PMC3543040

[B31] Lamb C. A. Yoshimori T. Tooze S. A. (2013). The Autophagosome: Origins Unknown, Biogenesis Complex. Nat. Rev. Mol. Cel Biol 14, 759–774. 10.1038/nrm3696 24201109

[B32] Lavallard V. J. Meijer A. J. Codogno P. Gual P. (2012). Autophagy, Signaling and Obesity. Pharmacol. Res. 66, 513–525. 10.1016/j.phrs.2012.09.003 22982482

[B33] Li S. Zhou Y. Gu X. Zhang X. Jia Z. (2021). NLRX1/FUNDC1/NIPSNAP1-2 axis Regulates Mitophagy and Alleviates Intestinal Ischaemia/reperfusion Injury[J]. Cell Prolif 3, e12986. 10.1111/cpr.12986 PMC7941235 33432610

[B34] Li W. Yin L. Sun X. Wu J. Dong Z. Hu K. (2020). Alpha-lipoic Acid Protects against Pressure Overload-Induced Heart Failure via ALDH2-dependent Nrf1-FUNDC1 Signaling. Cell Death Dis 11, 599. 10.1038/s41419-020-02805-2 32732978 PMC7393127

[B35] Li Y. Liu Z. Zhang Y. Zhao Q. Wang X. Lu P. (2018). PEDF Protects Cardiomyocytes by Promoting FUNDC1-mediated M-itophagy via PEDF-R under H-ypoxic C-ondition. Int. J. Mol. Med. 41, 3394–3404. 10.3892/ijmm.2018.3536 29512692 PMC5881750

[B36] Li Y. Xue Y. Xu X. Wang G. Liu Y. Wu H. (2019). A Mitochondrial FUNDC1/HSC70 Interaction Organizes the Proteostatic Stress Response at the Risk of Cell Morbidity. Embo j 38. 10.15252/embj.201798786 PMC6356068 30591555

[B37] Li Y. z. Wu X. d. Liu X. h. Li P. f. (2019). Mitophagy Imbalance in Cardiomyocyte Ischaemia/reperfusion Injury. Acta Physiol. 225, e13228. 10.1111/apha.13228 30507035

[B38] Lim Y. Berry B. Viteri S. McCall M. Park E. C. Rongo C. (2021). FNDC-1-mediated Mitophagy and ATFS-1 Coordinate to Protect against Hypoxia-Reoxygenation. Autophagy 17, 3389–3401. 10.1080/15548627.2021.1872885 33416042 PMC8632273

[B39] Liu B.-y. Li L. Liu G.-l. Ding W. Chang W.-g. Xu T. (2021). Baicalein Attenuates Cardiac Hypertrophy in Mice via Suppressing Oxidative Stress and Activating Autophagy in Cardiomyocytes. Acta Pharmacol. Sin 42, 701–714. 10.1038/s41401-020-0496-1 32796955 PMC8115069

[B40] Liu L. Li Y. Wang J. Zhang D. Wu H. Li W. (2021). Mitophagy Receptor FUNDC1 Is Regulated by PGC-1α/NRF1 to fine Tune Mitochondrial Homeostasis. EMBO Rep. 22, e50629. 10.15252/embr.202050629 33554448 PMC7926232

[B41] Liu L. Feng D. Chen G. Chen M. Zheng Q. Song P. (2012). Mitochondrial Outer-Membrane Protein FUNDC1 Mediates Hypoxia-Induced Mitophagy in Mammalian Cells. Nat. Cel Biol 14, 177–185. 10.1038/ncb2422 22267086

[B42] Liu L. Sakakibara K. Chen Q. Okamoto K. (2014). Receptor-mediated Mitophagy in Yeast and Mammalian Systems. Cell Res 24, 787–795. 10.1038/cr.2014.75 24903109 PMC4085769

[B43] Liu X. Ye B. Miller S. Yuan H. Zhang H. Tian L. (2012). Ablation of ALCAT1 Mitigates Hypertrophic Cardiomyopathy through Effects on Oxidative Stress and Mitophagy. Mol. Cel Biol 32, 4493–4504. 10.1128/mcb.01092-12 PMC3486149 22949503

[B44] Lv M. Wang C. Li F. Peng J. Wen B. Gong Q. (2017). Structural Insights into the Recognition of Phosphorylated FUNDC1 by LC3B in Mitophagy. Protein Cell 8, 25–38. 10.1007/s13238-016-0328-8 27757847 PMC5233613

[B45] Ma K. Zhang Z. Chang R. Cheng H. Mu C. Zhao T. (2020). Dynamic PGAM5 Multimers Dephosphorylate BCL-xL or FUNDC1 to Regulate Mitochondrial and Cellular Fate. Cell Death Differ 27, 1036–1051. 10.1038/s41418-019-0396-4 31367011 PMC7206082

[B46] Ma S. Chen J. Feng J. Zhang R. Fan M. Han D. (2018). Melatonin Ameliorates the Progression of Atherosclerosis via Mitophagy Activation and NLRP3 Inflammasome Inhibition[J]. Oxid Med. Cel Longev 2018, 9286458. 10.1155/2018/9286458 PMC6142770 30254716

[B47] Mao C. Hu C. Zhou Y. Zou R. Li S. Cui Y. (2020). Electroacupuncture Pretreatment against Cerebral Ischemia/Reperfusion Injury through Mitophagy. Evid. Based Complement. Alternat Med. 2020, 7486041. 10.1155/2020/7486041 32963572 PMC7499311

[B48] Mao S. Tian S. Luo X. Zhou M. Cao Z. Li J. (2021). Overexpression of PLK1 Relieved the Myocardial Ischemia-Reperfusion Injury of Rats through Inducing the Mitophagy and Regulating the P-Ampk/fundc1 axis. Bioengineered 12, 2676–2687. 10.1080/21655979.2021.1938500 34115550 PMC8806532

[B49] Mishra P. Chan D. C. (2016). Metabolic Regulation of Mitochondrial Dynamics. J. Cel Biol 212, 379–387. 10.1083/jcb.201511036 PMC4754720 26858267

[B50] Nakamura M. Sadoshima J. (2018). Mechanisms of Physiological and Pathological Cardiac Hypertrophy. Nat. Rev. Cardiol. 15, 387–407. 10.1038/s41569-018-0007-y 29674714

[B51] Nguyen T. N. Padman B. S. Lazarou M. (2016). Deciphering the Molecular Signals of PINK1/Parkin Mitophagy. Trends Cel Biol. 26, 733–744. 10.1016/j.tcb.2016.05.008 27291334

[B52] Pecoraro M. Pinto A. Popolo A. (2019). Mitochondria and Cardiovascular Disease: A Brief Account. Crit. Rev. Eukaryot. Gene Expr. 29, 295–304. 10.1615/critreveukaryotgeneexpr.2019028579 31679291

[B53] Pei Z. Liu Y. Liu S. Jin W. Luo Y. Sun M. (2021). FUNDC1 Insufficiency Sensitizes High Fat Diet Intake-Induced Cardiac Remodeling and Contractile Anomaly through ACSL4-Mediated Ferroptosis. Metabolism 122, 154840. 10.1016/j.metabol.2021.154840 34331963

[B54] Peng X. Chen H. Li Y. Huang D. Huang B. Sun D. (2020). Effects of NIX‐mediated Mitophagy on ox‐LDL‐induced Macrophage Pyroptosis in Atherosclerosis. Cell Biol Int 44, 1481–1490. 10.1002/cbin.11343 32181963

[B55] Poole L. P. Macleod K. F. (2021). Mitophagy in Tumorigenesis and Metastasis. Cell. Mol. Life Sci. 78, 3817–3851. 10.1007/s00018-021-03774-1 33580835 PMC8259496

[B56] Qiao H. Ren H. Du H. Zhang M. Xiong X. Lv R. (2018). Liraglutide Repairs the Infarcted Heart: The Role of the SIRT1/Parkin/mitophagy Pathway. Mol. Med. Rep. 17, 3722–3734. 10.3892/mmr.2018.8371 29328405 PMC5802177

[B57] Ren J. Sun M. Zhou H. Ajoolabady A. Zhou Y. Tao J. (2020). FUNDC1 Interacts with FBXL2 to Govern Mitochondrial Integrity and Cardiac Function through an IP3R3-dependent Manner in Obesity. Sci. Adv. 6. 10.1126/sciadv.abc8561 PMC7494344 32938669

[B58] Rogov V. V. Suzuki H. Marinković M. Lang V. Kato R. Kawasaki M. (2017). Phosphorylation of the Mitochondrial Autophagy Receptor Nix Enhances its Interaction with LC3 Proteins. Sci. Rep. 7, 1131. 10.1038/s41598-017-01258-6 28442745 PMC5430633

[B59] Shao D. Kolwicz S. C. Jr. Wang P. Roe N. D. Villet O. Nishi K. (2020). Increasing Fatty Acid Oxidation Prevents High-Fat Diet-Induced Cardiomyopathy through Regulating Parkin-Mediated Mitophagy. Circulation 142, 983–997. 10.1161/circulationaha.119.043319 32597196 PMC7484440

[B60] Silva-Palacios A. Zazueta C. Pedraza-Chaverri J. (2020). ER Membranes Associated with Mitochondria: Possible Therapeutic Targets in Heart-Associated Diseases. Pharmacol. Res. 156, 104758. 10.1016/j.phrs.2020.104758 32200027

[B61] Swiader A. Nahapetyan H. Faccini J. D’Angelo R. Mucher E. Elbaz M. (2016). Mitophagy Acts as a Safeguard Mechanism against Human Vascular Smooth Muscle Cell Apoptosis Induced by Atherogenic Lipids. Oncotarget 7, 28821–28835. 10.18632/oncotarget.8936 27119505 PMC5045359

[B62] Tagaya M. Arasaki K. (2017). Regulation of Mitochondrial Dynamics and Autophagy by the Mitochondria-Associated Membrane. Adv. Exp. Med. Biol. 997, 33–47. 10.1007/978-981-10-4567-7_3 28815520

[B63] Thomas A. Marek-Iannucci S. Tucker K. C. Andres A. M. Gottlieb R. A. (2019). Decrease of Cardiac Parkin Protein in Obese Mice. Front. Cardiovasc. Med. 6, 191. 10.3389/fcvm.2019.00191 32039238 PMC6984192

[B64] Tian R. Colucci W. S. Arany Z. Bachschmid M. M. Ballinger S. W. Boudina S. (2019). Unlocking the Secrets of Mitochondria in the Cardiovascular System. Circulation 140, 1205–1216. 10.1161/circulationaha.119.040551 31769940 PMC6880654

[B65] Tong M. Saito T. Zhai P. Oka S.-i. Mizushima W. Nakamura M. (2019). Mitophagy Is Essential for Maintaining Cardiac Function during High Fat Diet-Induced Diabetic Cardiomyopathy. Circ. Res. 124, 1360–1371. 10.1161/circresaha.118.314607 30786833 PMC6483841

[B66] Van Camp G. (2014). Cardiovascular Disease Prevention. Acta Clinica Belgica 69, 407–411. 10.1179/2295333714y.0000000069 25176558

[B67] Vásquez-Trincado C. García-Carvajal I. Pennanen C. Parra V. Hill J. A. Rothermel B. A. (2016). Mitochondrial Dynamics, Mitophagy and Cardiovascular Disease. J. Physiol. 594, 509–525. 10.1113/jp271301 26537557 PMC5341713

[B68] Wang C. Dai X. Wu S. Xu W. Song P. Huang K. (2021). FUNDC1-dependent Mitochondria-Associated Endoplasmic Reticulum Membranes Are Involved in Angiogenesis and Neoangiogenesis. Nat. Commun. 12, 2616. 10.1038/s41467-021-22771-3 33972548 PMC8110587

[B69] Wang J. Toan S. Zhou H. (2020). New Insights into the Role of Mitochondria in Cardiac Microvascular Ischemia/reperfusion Injury. Angiogenesis 23, 299–314. 10.1007/s10456-020-09720-2 32246225

[B70] Wang J. Zhu P. Li R. Ren J. Zhou H. (2020). Fundc1-dependent Mitophagy Is Obligatory to Ischemic Preconditioning-Conferred Renoprotection in Ischemic AKI via Suppression of Drp1-Mediated Mitochondrial Fission. Redox Biol. 30, 101415. 10.1016/j.redox.2019.101415 31901590 PMC6940662

[B71] Wang X. (2001). The Expanding Role of Mitochondria in Apoptosis. Genes Dev. 15, 2922–2933. 11711427

[B72] Wang Y. Jasper H. Toan S. Muid D. Chang X. Zhou H. (2021). Mitophagy Coordinates the Mitochondrial Unfolded Protein Response to Attenuate Inflammation-Mediated Myocardial Injury. Redox Biol. 45, 102049. 10.1016/j.redox.2021.102049 34174558 PMC8246635

[B73] Wang Z. Xia P. Hu J. Huang Y. Zhang F. Li L. (2021). LncRNA MEG3 Alleviates Diabetic Cognitive Impairments by Reducing Mitochondrial-Derived Apoptosis through Promotion of FUNDC1-Related Mitophagy via Rac1-ROS Axis. ACS Chem. Neurosci. 12, 2280–2307. 10.1021/acschemneuro.0c00682 33843209

[B74] Wei X. Wei X. Lu Z. Li L. Hu Y. Sun F. (2020). Activation of TRPV1 Channel Antagonizes Diabetic Nephropathy through Inhibiting Endoplasmic Reticulum-Mitochondria Contact in Podocytes. Metabolism 105, 154182. 10.1016/j.metabol.2020.154182 32061660

[B75] Wu H. Wang Y. Li W. Chen H. Du L. Liu D. (2019). Deficiency of Mitophagy Receptor FUNDC1 Impairs Mitochondrial Quality and Aggravates Dietary-Induced Obesity and Metabolic Syndrome. Autophagy 15, 1882–1898. 10.1080/15548627.2019.1596482 30898010 PMC6844496

[B76] Wu H. Xue D. Chen G. Han Z. Huang L. Zhu C. (2014). The BCL2L1 and PGAM5 axis Defines Hypoxia-Induced Receptor-Mediated Mitophagy. Autophagy 10, 1712–1725. 10.4161/auto.29568 25126723 PMC4198357

[B77] Wu S. Lu Q. Ding Y. Wu Y. Qiu Y. Wang P. (2019). Hyperglycemia-Driven Inhibition of AMP-Activated Protein Kinase α2 Induces Diabetic Cardiomyopathy by Promoting Mitochondria-Associated Endoplasmic Reticulum Membranes *In Vivo* . Circulation 139, 1913–1936. 10.1161/circulationaha.118.033552 30646747 PMC6465113

[B78] Wu S. Lu Q. Wang Q. Ding Y. Ma Z. Mao X. (2017). Binding of FUN14 Domain Containing 1 with Inositol 1,4,5-Trisphosphate Receptor in Mitochondria-Associated Endoplasmic Reticulum Membranes Maintains Mitochondrial Dynamics and Function in Hearts *In Vivo* . Circulation 136, 2248–2266. 10.1161/circulationaha.117.030235 28942427 PMC5716911

[B79] Wu S. Zou M.-H. (2019). Mitochondria-associated Endoplasmic Reticulum Membranes in the Heart. Arch. Biochem. Biophys. 662, 201–212. 10.1016/j.abb.2018.12.018 30571967 PMC6345610

[B80] Wu W. Xu H. Wang Z. Mao Y. Yuan L. Luo W. (2015). PINK1-Parkin-Mediated Mitophagy Protects Mitochondrial Integrity and Prevents Metabolic Stress-Induced Endothelial Injury. PLoS One 10, e0132499. 10.1371/journal.pone.0132499 26161534 PMC4498619

[B81] Wu W. Li W. Chen H. Jiang L. Zhu R. Feng D. (2016). FUNDC1 Is a Novel Mitochondrial-Associated-Membrane (MAM) Protein Required for Hypoxia-Induced Mitochondrial Fission and Mitophagy. Autophagy 12, 1675–1676. 10.1080/15548627.2016.1193656 27314574 PMC5082786

[B82] Wu W. Lin C. Wu K. Jiang L. Wang X. Li W. (2016). FUNDC 1 Regulates Mitochondrial Dynamics at the ER -mitochondrial Contact Site under Hypoxic Conditions. Embo j 35, 1368–1384. 10.15252/embj.201593102 27145933 PMC4864280

[B83] Wu W. Tian W. Hu Z. Chen G. Huang L. Li W. (2014). ULK 1 Translocates to Mitochondria and Phosphorylates FUNDC 1 to Regulate Mitophagy. EMBO Rep. 15, 566–575. 10.1002/embr.201438501 24671035 PMC4210082

[B84] Xiao Y. Chen W. Zhong Z. Ding L. Bai H. Chen H. (2020). Electroacupuncture Preconditioning Attenuates Myocardial Ischemia-Reperfusion Injury by Inhibiting Mitophagy Mediated by the mTORC1-ULK1-FUNDC1 Pathway. Biomed. Pharmacother. 127, 110148. 10.1016/j.biopha.2020.110148 32344255

[B85] Xin T. Lu C. (2020). Irisin Activates Opa1-Induced Mitophagy to Protect Cardiomyocytes against Apoptosis Following Myocardial Infarction. Aging 12, 4474–4488. 10.18632/aging.102899 32155590 PMC7093202

[B86] Xiong W. Hua J. Liu Z. Cai W. Bai Y. Zhan Q. (2018). PTEN Induced Putative Kinase 1 (PINK1) Alleviates Angiotensin II-Induced Cardiac Injury by Ameliorating Mitochondrial Dysfunction. Int. J. Cardiol. 266, 198–205. 10.1016/j.ijcard.2018.03.054 29887448

[B87] Xu X. Hua Y. Nair S. Bucala R. Ren J. (2014). Macrophage Migration Inhibitory Factor Deletion Exacerbates Pressure Overload-Induced Cardiac Hypertrophy through Mitigating Autophagy. Hypertension 63, 490–499. 10.1161/hypertensionaha.113.02219 24366076 PMC3929844

[B88] Yang H.-x. Wang P. Wang N.-n. Li S.-d. Yang M.-h. (2021). Tongxinluo Ameliorates Myocardial Ischemia-Reperfusion Injury Mainly via Activating Parkin-Mediated Mitophagy and Downregulating Ubiquitin-Proteasome System. Chin. J. Integr. Med. 27, 542–550. 10.1007/s11655-019-3166-8 31227964

[B89] Yang M. Li C. Yang S. Xiao Y. Xiong X. Chen W. (2020). Mitochondria-Associated ER Membranes - the Origin Site of Autophagy. Front. Cel Dev. Biol. 8, 595. 10.3389/fcell.2020.00595 PMC7378804 32766245

[B90] Yang M. Linn B. S. Zhang Y. Ren J. (2019). Mitophagy and Mitochondrial Integrity in Cardiac Ischemia-Reperfusion Injury. Biochim. Biophys. Acta (Bba) - Mol. Basis Dis. 1865, 2293–2302. 10.1016/j.bbadis.2019.05.007 31100337

[B91] Yoo S. M. Jung Y. K. (2018). A Molecular Approach to Mitophagy and Mitochondrial Dynamics. Mol. Cell 41, 18–26. 10.14348/molcells.2018.2277 PMC5792708 29370689

[B92] Yu W. Sun S. Xu H. Li C. Ren J. Zhang Y. (2020). TBC1D15/RAB7-regulated Mitochondria-Lysosome Interaction Confers Cardioprotection against Acute Myocardial Infarction-Induced Cardiac Injury. Theranostics 10, 11244–11263. 10.7150/thno.46883 33042281 PMC7532681

[B93] Yu W. Xu M. Zhang T. Zhang Q. Zou C. (2019). Mst1 Promotes Cardiac Ischemia-Reperfusion Injury by Inhibiting the ERK-CREB Pathway and Repressing FUNDC1-Mediated Mitophagy. J. Physiol. Sci. 69, 113–127. 10.1007/s12576-018-0627-3 29961191 PMC10717665

[B94] Zeinvand-Lorestani M. Kalantari H. Khodayar M. J. Teimoori A. Saki N. Ahangarpour A. (2018). Dysregulation of Sqstm1, Mitophagy, and Apoptotic Genes in Chronic Exposure to Arsenic and High-Fat Diet (HFD). Environ. Sci. Pollut. Res. 25, 34351–34359. 10.1007/s11356-018-3349-4 30302732

[B95] Zhang W. Ren H. Xu C. Zhu C. Wu H. Liu D. (2016). Hypoxic Mitophagy Regulates Mitochondrial Quality and Platelet Activation and Determines Severity of I/R Heart Injury[J]. Elife 5, e21407. 10.7554/eLife.21407 27995894 PMC5214169

[B96] Zhang X. Sergin I. Evans T. D. Jeong S.-J. Rodriguez-Velez A. Kapoor D. (2020). High-protein Diets Increase Cardiovascular Risk by Activating Macrophage mTOR to Suppress Mitophagy. Nat. Metab. 2, 110–125. 10.1038/s42255-019-0162-4 32128508 PMC7053091

[B97] Zhang Y. Sowers J. R. Ren J. (2018). Targeting Autophagy in Obesity: from Pathophysiology to Management. Nat. Rev. Endocrinol. 14, 356–376. 10.1038/s41574-018-0009-1 29686432

[B98] Zhou H. Zhang Y. Hu S. Shi C. Zhu P. Ma Q. (2017). Melatonin Protects Cardiac Microvasculature against Ischemia/reperfusion Injury via Suppression of Mitochondrial Fission-VDAC1-HK2-mPTP-Mitophagy axis. J. Pineal Res. 63. 10.1111/jpi.12413 PMC5518188 28398674

[B99] Zhou H. He L. Xu G. Chen L. (2020). Mitophagy in Cardiovascular Disease. Clinica Chim. Acta 507, 210–218. 10.1016/j.cca.2020.04.033 32360616

[B100] Zhou H. Wang J. Zhu P. Zhu H. Toan S. Hu S. (2018). NR4A1 Aggravates the Cardiac Microvascular Ischemia Reperfusion Injury through Suppressing FUNDC1-Mediated Mitophagy and Promoting Mff-Required Mitochondrial Fission by CK2α. Basic Res. Cardiol. 113, 23. 10.1007/s00395-018-0682-1 29744594

[B101] Zhou H. Zhu P. Guo J. Hu N. Wang S. Li D. (2017). Ripk3 Induces Mitochondrial Apoptosis via Inhibition of FUNDC1 Mitophagy in Cardiac IR Injury. Redox Biol. 13, 498–507. 10.1016/j.redox.2017.07.007 28732308 PMC5828768

[B102] Zhou H. Zhu P. Wang J. Zhu H. Ren J. Chen Y. (2018). Pathogenesis of Cardiac Ischemia Reperfusion Injury Is Associated with CK2α-Disturbed Mitochondrial Homeostasis via Suppression of FUNDC1-Related Mitophagy. Cel Death Differ 25, 1080–1093. 10.1038/s41418-018-0086-7 PMC5988750 29540794

[B103] Zimmermann M. Reichert A. S. (2017). How to Get Rid of Mitochondria: Crosstalk and Regulation of Multiple Mitophagy Pathways. Biol. Chem. 399, 29–45. 10.1515/hsz-2017-0206 28976890

